# Cases of Rheumatic Fever Treated by Salicylic Acid

**Published:** 1878-04

**Authors:** Alfred Kebble

**Affiliations:** M. R. C. S. England, Late House-Surgeon, York County Hospital


### MISCELLANEOUS.

### Cases of Rheumatic Fever Treated by Salicylic Acid.

BY ALFRED KEBBLE, M. R. C. S. England, Late House-Surgeon, York County Hospital.

CASE 1.—. C——, aged twenty-one. This was the third attack. On Jan. 9th, 1877, patient ran a mile to catch a train, and perspired freely. The day was very cold. She was a tall, stout girl, but of a sallow, unhealthy appearance. On the day following I saw her. She complained of not feeling well, of headache, pain in several joints, and a shortness of breathing. She kept about all day, but in the evening was compelled to go to bed. The temperature was then 105°. There was pain, swelling, and redness in both wrists and ankles; face flushed; some dullness at base of lungs posteriorly. Ordered milk and beef-tea diet, and twenty grains of salicylic acid was given every three hours.

Jan. 11th.--After taking about eight doses of the acid, the temperature is down to 101°; pain much less in the joints affected, but there is a delirium, with much depression; urine dark-colored; pain about region of the heart; pericardial friction-sound to be heard. Salicylic acid discontinued, and the usual remedies resorted to.

The patient continued to get worse, and died at the end of six days from pericarditis, accompanied by cerebral symptoms.

Case 2.—A. B——, aged thirty, cook. Has had rheumatic fever once before. I saw her on Jan. 14th, 1877. About a week before she began to have pain in several joints, accompanied by effusion. She now has effusion into both knee-joints, the right being the worst; also in both ankles and wrists. Has passed a very restless night; sweating freely; tongue furred. The records of the temperature were unfortunately not kept for the first few days of the illness. I prescribed fifteen grains of salicylic acid to be taken every three hours. The joints to be wrapped in cotton wool.

Jan. 15th.—Very much better; passed a good night; says she began to feel better after she had taken three or four doses of the medicine; effusion into knees nearly gone; can move around in bed—an impossibility yesterday. Although I do not remember the exact degree of temperature, I know that it was lowered about three degrees.

16th.—Still improving; mixture to be taken every six hours.

18th.—Has continued the mixture till to-day; seems almost well as far as the rheumatism is concerned; to take fifteen minims of tincture of perchloride of iron three times a day.

20th.—Going on well.

24th.—Return of the rheumatism; temperature 99.4°. To return to the acid mixture.

25th.—Temperature 98.4°; all the pain gone again; good night; sweated freely; complains of a peculiar feeling about the head and throat; vomited once this morning.

29th.—Slight return of pain in right shoulder and hip. Ten grains of the acid every three hours; eight doses given.

Feb. 2nd.—Continues better; to take the mixture twice a day.

5th.—Free from pain; mixture to be taken occasionally.

9th.—Quite well.

Case 3.—J. S.——, aged thirteen, school girl. First seen Feb. 11th, 1877. This is the first attack. Three days since she commenced to have pain in several joints. Temperature 102.6°. Right shoulder and wrist and both knees and ankles affected; great pain, which prevents her sleeping; she was screaming all night with the pain; cannot move herself in bed; no sweating. To take eight grains of salicylic acid every four hours.

Feb. 12th.—Passed a good night. Temperature 101°. Much easier. Can now turn herself in bed. Has turned herself right round in bed to speak to me. Left knee most painful, red and swollen. Continues mixture.

13th.—Temperature 98.4°. Very good night; almost free from pain.

15th.—Temperature 98.5°. Quite well. To get up to-day. Mixture to be taken if there is any return of the pain.

19th.—I called to see her, and found her down-stairs nursing the baby.

Case 4.—W. C.——, aged fifty-five, farmer. First seen June 28th, 1877. This is the fourth attack. Commenced a few days since with pain in the ankles. Temperature 101°; pulse 100. Both knees, ankles and wrists now affected. To take ten grains of salicylic acid every two hours. Cotton wool to joints. Ten grains of compound ipecacuanha powder at bedtime.

June 29th.—Temperature 101°. Restless night. Knees feel better. The patient is a very heavy man, weighing about seventeen stone. He is quite unable to move himself; lies with his feet over the end of the bed.

30th.—Temperature 99°; pulse 90. Passed a capital night. The patient and friends are much pleased at the sudden improvement. This morning he has been out of bed, and walked round the room. Mixture to be taken every three hours. Urine dark-colored.

July 1st—Not quite so good a night, but he had some friends to see him last evening, who stayed too long. Free from pain except in left hand and wrist. Mixture to be taken every six hours.

3d.—Going on well. Temperature 98°. Is very anxious to get up to-day. To sit up for a short time. Mixture to be taken three times a day.

4th.—Not quite so good a night. Slight return of pain in right shoulder.

5th.—Temperature 101°; pulse 90. Return of pain in both wrists, left knee and shoulders. Mixture to be taken every two hours.

6th.—Temperature 98°. Much better; good night. Mixture every two hours.

7th.—Temperature 99°. Going on well.

9th.—Temperature 100°; pulse 70; respiration 16. Passed a good night. Vomited once last evening after taking the medicine, and has not taken any since. Return of pain in left knee, over tendo-Achillis and left heel, right hand and shoulder. Tongue moist, slightly furred. Complains of sore throat and gums. Feels as though there were a lump at the back of the tongue. Complains of pain at pit of stomach. No symptoms of cardiac mischief. Eyes blood-shot; low-spirited. Bowels moved yesterday; urine natural in color and quantity. Up to this date I had been giving the acid in mucilage. I now altered it, and gave it with solution of acetate of ammonia and champhor-water.

10th.—Pulse 80; respiration 16; temperature 98°. Much better in all respects. Passed a good night. Now takes the mixture well

11th.—Temperature 99.2°. Feels low-spirited. Taking nourishment well.

13th.—Temperature 98.4°. Going on well.

16th.—Got up to-day. Quite free from pain. Mixture every four hours.

18th.—Up three hours. Mixture every six hours. Quite free from pain.

20th.—Mixture to be taken three times a day.

He continued the acid, in small doses, up to August 10th. He was very anxious not to leave it off too soon, as he had received so much benefit from it.

August 12th.—Free from rheumatism, but is not passing sufficient water—only one pint in twenty-four hours. Complains of pain in loins. Slight trace of albumen.

After this date the patient recovered his usual health, but occasionally having slight rheumatic pains. Although the symptoms of rheumatism have lasted about a month, the patient says that he has never been so free from pain in any previous attack. On the last occasion on which he suffered from rheumatic fever he was ill for about ten weeks, was unable to move himself in bed for a greater part of the time, and had to have relays of friends to move him night and day, on this occasion he was nursed by a daughter of fourteen years and one woman, who sat up with him at night. He was always able to get up to the night-stool.

Case 5.—J. E——, age forty-five, platelayer. Seen on June 15th, 1877. Has been ill about a fortnight. Has been very wet at his work lately. Temperature 100°. Has effusion into both knee-joints and ankles; has had pain in shoulders. To take fifteen grains of salicylic acid every three hours.

June 17th.—Much easier; can now turn in bed. Effusion into knees much less; that of ankles gone. Complains of deafness. Has a noise like that of a water-mill in his head. Has vomited twice after the medicine; to take it every six hours.

19th.—Temperature 98.4°. Mixture three times a day. From this date the patient relapsed into a state of chronic rheumatism. The salicylic acid was discontinued, as no further benefit seemed to be derived from its use; and the patient is at the present time —four months after the commencement of his illness—unable to work.

In the few cases that I have treated there has been rapid lowering of temperature with diminished pain, but there has been a tendency to depression with head symptoms, which, however, soon passed off on diminishing the quantity and frequency of the dose. I think it is necessary to continue the drug in small doses for some days after the departure of all symptoms of rheumatism, otherwise there is very likely to be a relapse.—*London Lancet*

